# Projected Reduction of Diabetes- and Obesity-Related Complication Risks Following the 12-Week Weight-Loss Phase of the RESET Study

**DOI:** 10.36469/001c.162856

**Published:** 2026-06-16

**Authors:** Bjoern Schwander, Kirk W. Kerr, Ricardo Rueda, Maria Camprubi-Robles, Nicky Northway, Carl Lumsden, Louise Taylor, Suela Sulo

**Affiliations:** 1 AHEAD GmbH, Bietigheim-Bissingen, Germany; 2 Abbott Nutrition, Columbus, Ohio, USA; 3 Abbott Nutrition, Granada, Spain; 4 Changing Health Ltd., London, UK

**Keywords:** cost-effectiveness analysis, weight-loss intervention, diabetes-related complications, risk-projection modeling, health economic modeling, type 2 diabetes

## Abstract

**Background:**

Type 2 diabetes (T2D) and obesity increase individuals’ risk of microvascular and macrovascular complications, which may increase healthcare costs. The RESET program combined low-calorie diet using diabetes-specific nutritional formula and a digital lifestyle behavior change program to target sustainable weight loss and improved diabetes management.

**Objective:**

To quantify projected changes in diabetes-related complication risks following the 12-week weight-loss phase of the RESET program and to relate these modeled risk reductions to program costs.

**Methods:**

Data from 157 adults with type 2 diabetes (mean age, 56 years; diabetes duration, 2.2 years; baseline body mass index (BMI), 35 kg/m²; HbA1c, 7.5%) completing the RESET weight-loss phase were analyzed. Observed mean changes in HbA1c, BMI, and systolic blood pressure were applied to the UK Prospective Diabetes Study Outcomes Model 2 (UKPDS-OM2) to estimate projected relative risk reductions in microvascular and macrovascular complications over a 3-month horizon. Program costs were derived using a microcosting approach from the healthcare payer perspective, and parameter uncertainty was evaluated through 20 000 Monte Carlo simulations.

**Results:**

Participants achieved mean reductions of 1.0% in HbA1c, 11.0 kg in body weight, and 4.5 mmHg in systolic blood pressure. The model projected an overall relative reduction in total complication risk of 15.4% (95% confidence interval [CI], 9.1-21.4), corresponding to an absolute reduction of 1.9 projected events per 1000 participants over 3 months, comprising a –13.2% mean reduction in macrovascular and –23.8% in microvascular complication risks. Mean program cost was £1236 per participant (95% CI, £1001-£1492), corresponding to an incremental cost of £84 per 1% relative risk reduction.

**Conclusions:**

Short-term, intensive weight loss achieved clinically meaningful improvements in HbA1c and body weight that were associated with favorable reductions in projected microvascular and macrovascular complications at modest cost. Absolute event reductions over 3 months were modest, and sustaining these improvements is essential to realize long-term clinical and economic benefit.

## INTRODUCTION

Type 2 diabetes is a major and growing public health challenge, contributing substantially to morbidity, premature mortality, and healthcare expenditures worldwide, with much of the burden driven by cardiovascular and microvascular complications.^1,2^ While pharmacologic therapy has expanded and improved glycemic management, many individuals continue to fail to achieve durable control, and disease progression frequently persists in real-world settings.^3,4^

Substantial weight loss and intensive lifestyle interventions have consistently been shown to improve glycemic control and, in some cases, induce remission of type 2 diabetes.^5–7^ Large clinical trials and mechanistic studies demonstrate that dietary interventions and structured lifestyle changes can reduce hemoglobin A1c (HbA1c), decrease the need for glucose-lowering medication, and improve cardiometabolic risk factors such as blood pressure and lipid levels.^5–7^ Collectively, these findings indicate that lifestyle-based interventions can provide benefits beyond glycemic control, with the potential to modify disease progression.

Despite these encouraging findings, most studies report surrogate outcomes such as HbA1c, body weight, blood pressure, and lipid levels. While these markers are clinically relevant, their direct translation into patient-centered outcomes, such as myocardial infarction, stroke, or progression of microvascular disease, remains uncertain. This gap limits the ability to fully assess the clinical impact of interventions aiming at remission.

Risk prediction models, such as the UK Prospective Diabetes Study (UKPDS) Outcomes Model 2, allow integration of multiple risk factor changes to estimate the projected risk of vascular complications.^8–10^ Diabetes simulation models have commonly been used in health economic evaluations to extrapolate short- to medium-term changes in HbA1c, body weight, blood pressure, and other risk factors to long-term complications, costs, and quality-adjusted survival.^10–13^ However, applications of such models to short-term lifestyle or remission-program data remain limited, particularly where the objective is to describe short-horizon projected complication risk rather than lifetime cost-effectiveness.

The aim of this study was to quantify the extent to which improvements in glycemic control and other cardiometabolic risk factors translate into projected changes in the risk of diabetes-related microvascular and macrovascular complications, and to relate these projected risk changes to the costs of the intervention program.

## METHODS

### Study Design and Population

Data for this analysis were derived from the RESET program, a pragmatic, multisite weight-loss intervention delivered in UK primary care. The present work focuses on participants in the 12-week weight-loss phase. Although the RESET program included subsequent transition and maintenance phases, the present analysis was restricted to the initial 12-week weight-loss phase because this was the predefined phase for the current risk-projection analysis. Details on study design, participating practices (28 in Northeast England), eligibility criteria (age 20-70 years, type 2 diabetes ≤6 years, body mass index [BMI] 27-45 kg/m², and exclusions), recruitment and consent procedures, and program components have been reported previously.^14,15^ The evaluation was conducted as a National Health Service (NHS) clinical service audit and was registered at ClinicalTrials.gov (NCT05483140).

### Data Source and Measurements

Clinical data were available from routine care records at baseline and after 12 weeks, including body weight (and BMI), HbA1c (reported as % and mmol/mol), and blood pressure (systolic/diastolic). When clinic measured weight was unavailable, validated self-reported values were used, as specified in the RESET protocol. Participants without either clinic-measured or validated self-reported weight data at the relevant assessment time point were excluded from analyses requiring weight-related inputs. The dietary intervention targeted approximately 900-1000 kcal/day with options including diabetes-specific nutrition formula meal replacements; coaching and digital self-management components followed a standardized protocol. Full details of measurement procedures, intervention delivery, and adherence definitions have been described elsewhere.^14,15^

### Modeling Approach

This analysis applied a cohort state-transition modeling framework based on risk equations from the UK Prospective Diabetes Study Outcomes Model 2 (UKPDS-OM2).^10^ In brief, the model estimated transitions from the baseline diabetes state to diabetes-related macrovascular events, microvascular events, or death over the modeled time horizon. Event-specific transition probabilities were derived from UKPDS-OM2 risk equations using patient characteristics and risk-factor values. The present analysis compared projected event probabilities before and after applying the observed 12-week changes in HbA1c, BMI, and systolic blood pressure. More broadly, the UKPDS-OM2 predicts the occurrence of major diabetes-related microvascular and macrovascular complications using risk factors such as age, sex, duration of diabetes, HbA1c, blood pressure, BMI, and lipid levels.

For this analysis, observed mean changes in HbA1c, BMI, and systolic blood pressure over 12 weeks, together with their confidence intervals (CI), were entered as model inputs. Risk projections were adjusted to a 3-month time horizon, and reductions were estimated by comparing projected complication risk at baseline values with risk recalculated using post-intervention values. These reductions are expressed as relative risk reductions (%), calculated as the proportional decrease from baseline to post-intervention. Corresponding absolute event risks over the 3-month horizon are reported separately.

Because UKPDS-OM2 risk equations are conventionally applied over annual cycles, 3-month event probabilities were derived by converting annual event probabilities to quarterly probabilities under a constant-hazard assumption. Specifically, annual probabilities were transformed using *p_3m_* = 1 – (1 – *p_annual_*)^0.25^. As described herein, the resulting estimates represent short-horizon, model-derived event probabilities rather than observed complication incidence.

Uncertainty was addressed using Monte Carlo simulations, which generated CIs around the estimated risk reductions. The UKPDS-OM2 has undergone external validation and is widely applied in diabetes outcomes research.^10,16,17^

### Outcomes

The primary outcome was the projected relative risk reduction (%) in combined risk of diabetes-related microvascular and macrovascular complications, estimated by applying the UKPDS-OM2 to the RESET study baseline and post-intervention values.

Secondary outcomes included the projected relative risk reductions of individual macrovascular events (myocardial infarction, ischemic heart disease, stroke, congestive heart failure) and microvascular events (amputation, blindness, renal failure, and diabetic ulcer).

Economic outcomes comprised the incremental cost of the intervention program and, as a proxy measure of cost-effectiveness, the incremental cost per 1% relative reduction in combined complication risk.

### Costing Approach

The costing analysis was conducted from the healthcare payer perspective. Only direct program costs for the RESET weight-loss intervention were included, covering dietary products, coaching, and digital support. No downstream costs of complications were incorporated, as the purpose of this analysis was to relate program expenditures to changes in projected complication risk.

To provide a realistic estimate of fixed costs per participant, the analysis assumed rollout of the program to approximately 1000 individuals with type 2 diabetes in the United Kingdom. This assumption reflects a feasible scale for population-level implementation and allows fixed program costs to be distributed across a larger patient base. Average program costs were then calculated per participant over the 12-week intervention and expressed in 2025 UK pounds sterling.

### Statistical Analysis

Analyses presented in this manuscript were restricted to participants completing the 12-week weight-loss phase with available observed outcome data. No imputation of missing data was performed. Accordingly, sample sizes varied across outcomes depending on data availability. Results should therefore be interpreted as observed-case analyses of Phase 1 completers.

Baseline values for all risk factors included in the UKPDS-OM2 were modeled as normally distributed using observed means and standard deviations from the RESET study. Among these, observed changes were applied to HbA1c, BMI, and systolic blood pressure, expressed as incremental changes from baseline to the end of the 12-week weight-loss phase (eg, mean reduction in body weight of –10.9 kg; standard deviation [SD] 6.6). For each simulation, baseline distributions were combined with observed change distributions to generate post-intervention values.

Uncertainty was evaluated using probabilistic sensitivity analysis with 20 000 Monte Carlo iterations. Convergence was confirmed using three independent runs with different random seeds; variation in run-level means for the primary combined outcome was <0.05 percentage points, and 95% CIs were highly stable across runs. Program costs were modeled using gamma distributions, with an assumed ±10% variation around estimated mean costs to reflect expected uncertainty in resource use and pricing. Total costs and corresponding 95% CIs were derived from these simulations. The model was programmed in Microsoft Excel 365 (Microsoft Corp.).

## RESULTS

### Study Design and Population

A total of 157 participants completed the weight-loss phase of the RESET program and were included in the present analysis.^15^ Baseline characteristics are shown in **Table 1**. The mean age was 56 years, mean BMI was 35 kg/m², mean HbA1c was 7.5% (58 mmol/mol), and mean systolic blood pressure was 135 mmHg. The mean duration of type 2 diabetes was 2.2 years. Forty-five percent of participants were women, and 28% reported an ethnicity other than White. At baseline, 36% of participants were not on glucose-lowering medication, 53% used 1 agent, and 8% used 2 or more agents.

**Table 1.** Baseline Characteristics of RESET Participants Completing the Weight-Loss Phase (N = 157)

**Characteristic**	**Mean ± SD or n (%)**	**Source**
Age, years	56 ± 9	RESET
Female sex	71 (45%)	RESET
Ethnicity, White	113 (72%)	RESET
Ethnicity, other	44 (28%)	RESET
Time since diabetes diagnosis, years	2.2 ± 1.8	RESET
HbA1c, % (mmol/mol)	7.5 ± 1.0 (58 ± 11)	RESET
BMI, kg/m²	35 ± 5	RESET
Systolic blood pressure, mmHg	135 ± 15	RESET
Diastolic blood pressure, mmHg	83 ± 8	RESET
Antidiabetic medication use		RESET
0	57 (36%)	RESET
1	83 (53%)	RESET
≥2	13 (8%)	RESET
DSNF meal replacements/day		RESET
0	2 (1%)	RESET
2	26 (17%)	RESET
4	129 (82%)	RESET
Total cholesterol, mmol/L	3.4 ± 1.0	UKPDS
HDL cholesterol, mmol/L	1.1 ± 0.3	UKPDS
Current smoker	21%	UKPDS

Abbreviations: BMI, body mass index; DSNF, diabetes-specific nutrition formula; HbA1c, hemoglobin A1c; HDL, high-density lipoprotein; UKPDS, UK Prospective Diabetes Study.

For variables required to parameterize the UKPDS-OM2 but not collected in RESET (eg, lipid levels, smoking status, renal function, cardiovascular history), values were supplemented from the UKPDS cohort data.^9,18,19^ These supplemented inputs were used solely for model parameterization and did not affect observed RESET outcomes.

### Risk Factor Changes at 12 Weeks

At 12 weeks, participants completing the weight-loss phase achieved substantial improvements in cardiometabolic outcomes, as presented in **Table 2.**^15^ Mean (SD) body weight decreased by 11.0 kg (6.5), corresponding to a BMI reduction of 3.7 (2.2) kg/m². HbA1c declined by 10.7 (12.2) mmol/mol (1.0% [1.1]). Systolic blood pressure was reduced by 4.5 (16.1) mmHg, and diastolic blood pressure by 5.8 (16.9) mmHg. All changes were statistically significant compared with baseline.

**Table 2.** Change in Cardiometabolic Outcomes at 12 Weeks (RESET Completers)

**Outcome**	**n**	**Mean (SD) Change**	***P* Value vs Baseline**
Weight (kg)	127	–11.0 (6.5)	<.001
BMI (kg/m²)	127	–3.7 (2.2)	<.01
HbA1c (mmol/mol)	111	–10.7 (12.2)	<.001
HbA1c (%)	111	–1.0 (1.1)	<.001
Systolic BP (mmHg)	102	–4.5 (16.1)	.005
Diastolic BP (mmHg)	102	–5.8 (16.9)	.006

Abbreviations: BMI, body mass index; BP, blood pressure; HbA1c, hemoglobin A1c; HDL, high-density lipoprotein; SD, standard deviation.

Values are mean change (SD) from baseline. *P* values for change from baseline. Data from RESET weight-loss phase completers.^15^

RESET cohort values were taken from baseline assessments of weight-loss phase completers.^15^ Supplemented values (lipids, smoking) were obtained from the UKPDS cohort^9,18,19^ (Leal et al, 2021) to allow full parameterization of the UKPDS-OM2.

It should be noted that only three risk factors — BMI, HbA1c, and systolic blood pressure — were available from RESET to inform the UKPDS-OM2 projections.^10^ These factors contribute differently across the model’s event equations. For example, changes in BMI influenced coronary heart failure projections, whereas both HbA1c and systolic blood pressure contributed to estimated risks of myocardial infarction and stroke. Similarly, microvascular complications such as amputation and blindness were influenced primarily by HbA1c and blood pressure, while ulcer risk was driven by BMI and HbA1c. As a result, projected risk reductions vary by complication and are likely to represent conservative estimates, since other risk factors included in the UKPDS-OM2 (eg, lipids, smoking, renal function) were not assessed in the RESET study.

### Projected Risk Reductions

**Figure 1.**
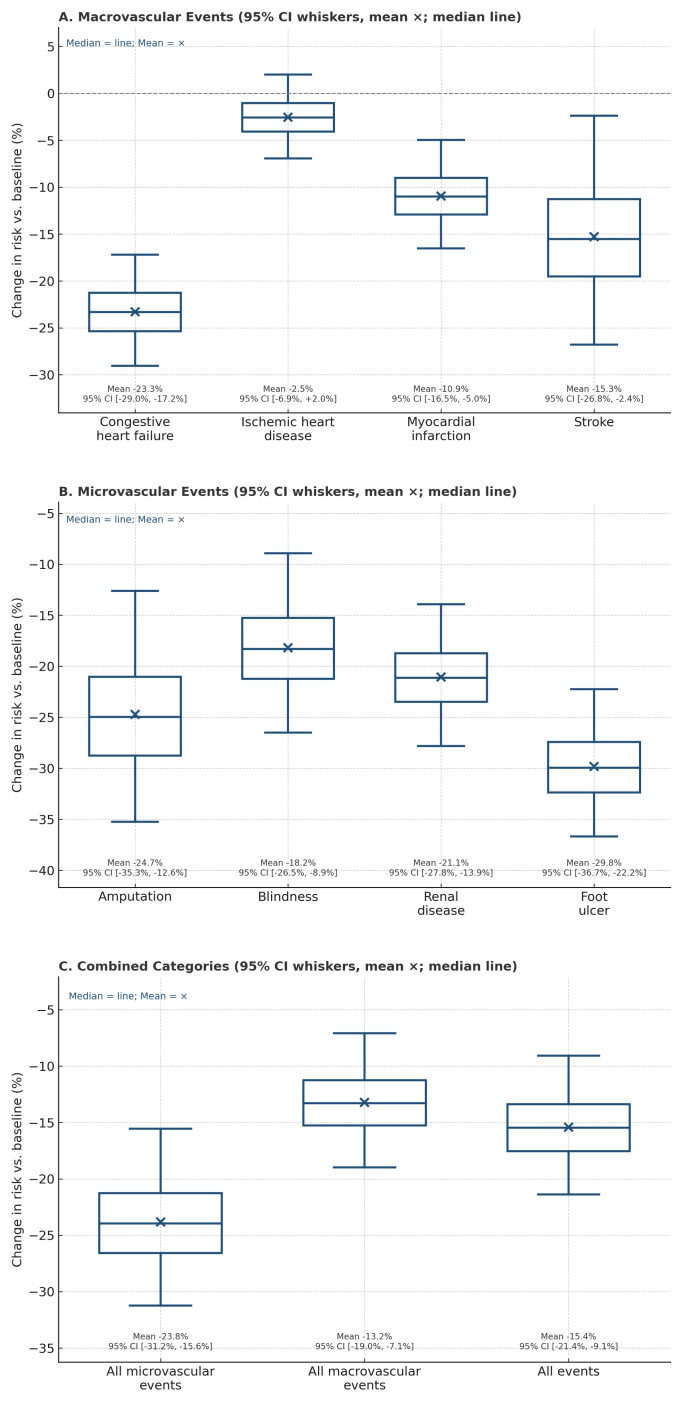
Projected Relative Risk Reduction in Diabetes-Related Complications Boxplots display the distribution of relative risk reduction (%) across 20,000 probabilistic simulations. Boxes represent the interquartile range, horizontal lines indicate medians, and crosses denote means. Whiskers correspond to the 95% confidence interval (2.5th to 97.5th percentiles). (**A**) Macrovascular events; (**B**) microvascular events; (**C**) combined outcomes.

Using the UKPDS-OM2 model, the RESET intervention was projected to yield consistent relative risk reductions in diabetes-related complications at the end of the 12-week weight-loss phase. **Figure 1** presents the distribution of relative risk reductions across 20 000 probabilistic simulations, shown separately for macrovascular events (Panel A), microvascular events (Panel B), and combined categories (Panel C).

For macrovascular events, relative risk reductions were most pronounced for congestive heart failure (mean, –23.3%; 95% CI, –29.1% to –17.2%) and stroke (–15.3%; 95% CI, –26.8% to –2.4%). Myocardial infarction decreased by –10.9% (95% CI, –16.5% to –5.0%), whereas ischemic heart disease showed only a modest relative decline (–2.5%; 95% CI, –6.9% to +2.0%).

For microvascular events, reductions were substantial across all endpoints: amputation (–24.7%; 95% CI, –35.3% to –12.6%), foot ulcer (–29.8%; 95% CI, –36.7% to –22.2%), blindness (–18.2%; 95% CI, –26.5% to –8.9%), and renal disease (–21.1%; 95% CI, –27.8% to –13.9%).

When aggregated across categories, the intervention was associated with an overall mean relative risk reduction of –15.4% (95% CI, –21.4% to –9.1%).

It is important to note that these values represent relative risk reductions. The corresponding absolute event risks over the 3-month horizon are presented in **Supplementary Table S1**.

When aggregated across outcomes, the mean projected risk of any macrovascular event decreased from 8.2 to 7.1 events per 1000 patients (absolute reduction, 1.1 events). For microvascular outcomes, the combined risk fell from 2.3 to 1.8 events per 1000 patients (absolute reduction, 0.6 events). Taken together, the total projected event risk decreased from 12.0 to 10.1 events per 1000 patients, corresponding to 1.9 fewer events per 1000 patients over 3 months. These absolute numbers are small given the short observation window, underscoring the importance of sustaining the observed improvements in risk factors over longer time horizons, since relapse toward baseline risk levels would diminish the long-term benefits.

### Program Costs

The mean cost of delivering the RESET intervention during the 12-week weight-loss phase was estimated at £1236 per participant (95% CI, £1112-1360), based on a microcosting approach that included both fixed and variable components. Fixed setup and infrastructure costs (NHS setup, £100 000; program build, £175 000; total, £275 000) were annualized and allocated assuming rollout to 1000 participants, equivalent to £22.92 per participant per month, or £69 per participant during the weight-loss phase. Variable costs consisted primarily of nutrition products (£942 per participant for 270 units of Glucerna®), with unit costs provided by the manufacturer (Abbott Nutrition), and service delivery costs for coaching and digital platform support (£225 per participant for 3 months), provided by Changing Health, the firm that coordinated the RESET study. All estimates incorporated a ±10% uncertainty range to reflect possible variation in resource use and unit costs. The mean total cost per participant (£1236) reflects the combined contribution of fixed and variable components, with uncertainty propagated through the probabilistic sensitivity analysis (±10% variation applied to each cost input).

### Cost Effectiveness

Probabilistic sensitivity analysis (20 000 simulations) showed that the RESET intervention achieved a mean projected relative risk reduction of 15.4% (95% CI, 9.1%-21.4%) during the 12-week weight-loss phase, at a mean cost of £1236 per participant (95% CI, £1001-£1492). The corresponding incremental cost-effectiveness ratio, expressed as the cost per 1% reduction in projected overall event risk, was £84.25 (median, £79.94; 95% CI, £54.17-£138.89).

**Figure 2.**
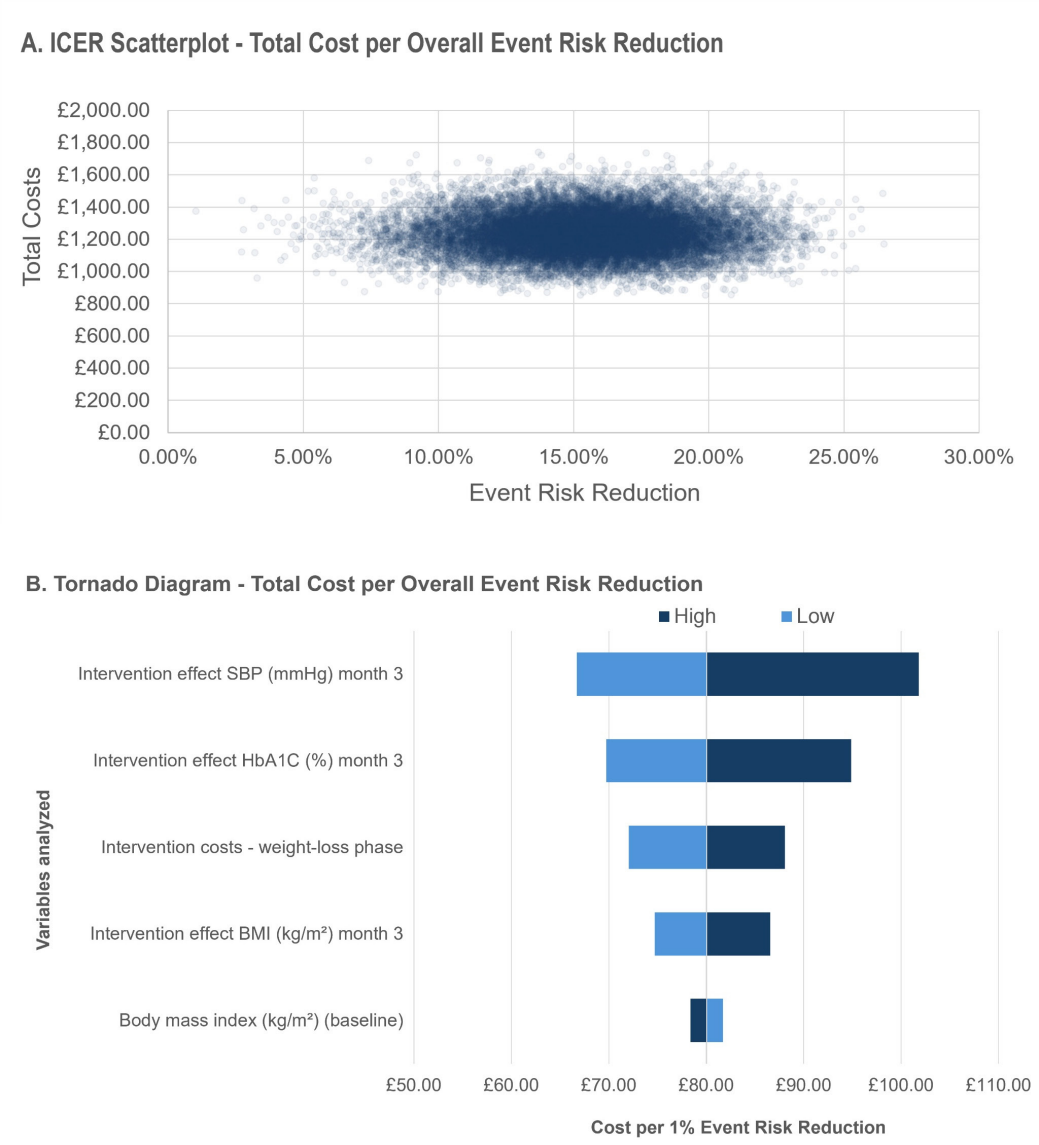
Cost-Effectiveness Analysis of the RESET Intervention Abbreviations: BMI, body mass index; HbA1c, hemoglobin A1c; ICER, incremental cost-effectivness ratio; SBP, systolic blood pressure. (**A**) Incremental cost-effectiveness scatterplot, displaying 20 000 probabilistic simulations of total cost per participant and relative reduction in overall event risk; (**B**) tornado diagram, showing the relative impact of key cost and outcome parameters on the cost per 1% reduction in overall event risk.

The scatterplot of incremental costs against event risk reduction (**Figure 2A**) illustrates the spread of simulated results. Nearly all simulations cluster within the cost range of £1000-£1500 and event risk reductions between 9% and 21%, confirming the robustness of the findings.

Deterministic sensitivity analysis highlighted the key drivers of cost-effectiveness (**Figure 2B**). The most influential parameters were the intervention effect on systolic blood pressure and HbA1c at month 3, followed by total intervention costs and the intervention effect on BMI. Baseline characteristics such as BMI, HDL-cholesterol, and LDL-cholesterol contributed minimally to variation in incremental cost-effectiveness ratio values.

## DISCUSSION

The RESET Phase 1 economic evaluation translated short-term improvements in HbA1c, body weight, and systolic blood pressure into projected relative risk reductions in diabetes-related complications using the UKPDS-OM2 model. Over the 12-week weight-loss phase, these changes produced consistent proportional decreases across both macrovascular and microvascular outcomes, with a mean overall relative risk reduction of approximately 15%, corresponding to 1.9 fewer projected events per 1000 participants over 3 months. Given the short time horizon, absolute event reductions were necessarily modest and should be interpreted alongside the relative risk reductions. In parallel, short-horizon cost-effectiveness analysis indicated favorable efficiency, with an average incremental cost of about £84 per 1% relative reduction in overall complication risk. Because the analytic window was limited to 3 months, absolute event risks were low, underscoring that the clinical and economic relevance of these findings depends on maintaining the observed improvements in glycemic control, body weight, and blood pressure over longer periods.

The projected relative risk reductions observed in this analysis align with the physiological pathways through which intensive weight-loss and metabolic improvement reduce cardiovascular and microvascular risk in type 2 diabetes.^8,10,20^ Similar magnitudes of change have been documented in structured lifestyle and remission programs such as the Diabetes Remission Clinical Trial (DiRECT)^6,21,22^ and the Look AHEAD study,^5,23^ where reductions in body weight and HbA1c of comparable size were associated with improvements in cardiometabolic profiles and, over time, lower incidence of diabetes-related complications. The present modeling extends these empirical observations by quantifying their potential impact on predicted complication risk using a validated, population-based tool.

The UK Prospective Diabetes Study Outcomes Model 2 (UKPDS-OM2) has been extensively validated and is widely used to project diabetes outcomes from short-term changes in surrogate risk factors.^10,17^ It is also recommended by the National Institute for Health and Care Excellence (NICE) as a preferred modeling framework for estimating diabetes-related complications and long-term outcomes in economic evaluations.^11^ Most prior diabetes modeling studies have used short- or medium-term intervention effects as inputs for long-term or lifetime projections of complications, costs, and quality-adjusted survival.^12,13^ The present analysis differs by focusing on short-horizon projected event probabilities during the observed 12-week weight-loss phase rather than estimating lifetime cost-effectiveness. This approach provides an exploratory translation of early concurrent improvements in HbA1c, systolic blood pressure, and body weight into modeled complication-risk terms.

The magnitude of these observed changes also meets established benchmarks for clinical relevance. A reduction of approximately 1 percentage point in HbA1c exceeds the threshold typically used to define meaningful improvement in glycemic control and has been linked in long-term cohort analyses to markedly lower risks of microvascular and macrovascular complications.^24–26^ Likewise, an average weight loss of about 10 kg—usually equivalent to ≥5 % and often approaching 10% of baseline body weight—corresponds to levels consistently associated with remission of hyperglycemia and improvement in cardiometabolic parameters.^5,22,27^ These changes therefore provide a strong mechanistic basis for the modeled relative risk reductions generated by the UKPDS-OM2 simulations.

Although the analytic time horizon of 3 months limits inference about long-term event prevention, the direction and magnitude of the modeled effects are biologically plausible and consistent with the evidence base showing that achieving a ≥1% HbA1c decrease and ≥5%-10% weight loss confers substantial health benefit if maintained. In this sense, the modeling results provide early proof-of-concept that clinically meaningful short-term improvements in metabolic risk factors can translate into measurable relative reductions in projected complication risk, while emphasizing that sustained maintenance of these improvements is essential for realizing their full clinical potential.

From a cost-effectiveness perspective, the short-horizon analysis demonstrates that achieving measurable improvements in glycemic control and weight during the 12-week program can be accomplished at modest incremental cost. The probabilistic sensitivity analysis produced a mean incremental cost of approximately £84 per 1% relative reduction in overall complication risk, with narrow uncertainty bounds across 20,000 simulations. This framing allows the efficiency of the weight-loss phase to be expressed independently of any assumed long-term horizon and provides a pragmatic benchmark for early-phase decision making.

While most published economic evaluations of diabetes interventions report incremental cost-effectiveness ratios in terms of cost per quality-adjusted life-year (QALY) gained,^12,13,28^ such analyses typically require long-term extrapolation of treatment effects and associated costs. The present analysis adopts a deliberately conservative time horizon to link short-term metabolic benefits directly to projected reductions in complication risk. This approach is consistent with recommendations for early-phase or proof-of-concept evaluations, which emphasize the value of quantifying short-term efficiency before undertaking full lifetime modeling.^29,30^

The probabilistic framework also illustrates the robustness of findings to parameter uncertainty. Variation in the ICER across simulations was primarily driven by the magnitude of intervention effects on HbA1c and systolic blood pressure, with smaller contributions from assumptions regarding program costs and body-mass-index change—patterns consistent with previous sensitivity analyses in diabetes models.^12,31^ These results suggest that even within plausible limits of variability, the intervention maintains a favorable short-term cost-effectiveness profile. The interpretation, however, should remain cautious because absolute event rates over three months are extremely low and downstream cost offsets were not modeled. The reported ratios are intended only as short-term indicators of efficiency, not as substitutes for lifetime cost-utility estimates.

Although the present evaluation focused on a short, 12-week horizon, the pattern of improvement observed has broader clinical and policy relevance. Achieving early and substantial reductions in HbA1c and body weight is known to improve the likelihood of diabetes remission and to delay or prevent progression of microvascular and macrovascular complications if such benefits are sustained.^5,22,27^ The results therefore reinforce the clinical importance of maintaining weight loss and glycemic control beyond the initial intervention period. Evidence from longitudinal cohorts shows that relapse of body weight and metabolic parameters toward baseline levels typically leads to rapid loss of these benefits, highlighting the need for ongoing structured support and relapse-prevention strategies in routine care.^22,23,32^ Longer-term RESET follow-up data from the transition and maintenance phases were not included in the present analysis and will be needed to assess whether the projected risk reductions persist, diminish, or increase over time.

From a service-delivery perspective, the modeled findings provide quantitative evidence to support the inclusion of structured weight-management and digital coaching programs within primary-care pathways for people with early type 2 diabetes. The intervention costs identified here—approximately £1200 per participant—are modest relative to the long-term costs associated with diabetes complications and are comparable to existing lifestyle or pharmacologic programs implemented within the NHS.^33^ If longer-term benefits are sustained, integrating such programs at scale may represent a potentially cost-efficient component of comprehensive diabetes management, particularly if mechanisms are in place to maintain engagement and weight stability over time.

Finally, these results underscore the value of using validated risk-projection tools such as the UKPDS-OM2 within quality-improvement and commissioning frameworks. Linking short-term clinical outcomes to modeled risk reduction provides health systems with a transparent method to estimate downstream benefit and to prioritize interventions that deliver the greatest potential return on investment in population health terms.

The major strength of this analysis lies in the use of empirical data from a real-world, primary-care weight-loss program combined with a validated and externally calibrated outcomes model. By applying the UKPDS-OM2 to observed short-term changes in HbA1c, body weight, and systolic blood pressure, the modeling captured the joint influence of multiple modifiable risk factors on predicted diabetes-related events. The use of probabilistic sensitivity analysis with 20 000 Monte Carlo iterations provides a robust quantification of parameter uncertainty, while convergence testing confirmed the stability of simulated results. Together, these methodological steps strengthen confidence in the internal validity and reproducibility of the findings.

Nonetheless, several limitations should be acknowledged. First, the 3-month time horizon restricts interpretation to short-term, relative and absolute changes in modeled risk rather than actual event prevention. Absolute event probabilities during this period are extremely low, meaning that the absolute risk reductions are necessarily small and sensitive to baseline risk assumptions. Second, no downstream medical-cost offsets or QALY gains were included; the resulting “cost per 1% relative risk reduction” should therefore be interpreted as a short-term efficiency metric rather than a conventional cost-utility ratio. Third, analyses were restricted to participants completing Phase 1 with available observed data, and no imputation procedures were applied. The present analysis also did not formally compare baseline characteristics between completers and noncompleters or between participants with and without available follow-up measurements. As a result, the potential impact of attrition and incomplete outcome ascertainment on projected risk estimates could not be quantified. Fourth, the analysis assumed that mean changes in HbA1c, weight, and blood pressure were normally distributed and independent. Patient-level covariance estimates among these changes were not available for the present analysis; therefore, probabilistic simulations did not account for potential correlations between risk-factor changes. This may have influenced the estimated uncertainty surrounding projected outcomes. Fifth, the UKPDS-OM2 was developed in a UK population of newly diagnosed diabetes patients, and although its equations have been validated across multiple cohorts, some calibration uncertainty may remain when applied to contemporary populations or to individuals with shorter diabetes duration.

Finally, the cost analysis was limited to program-delivery costs, excluding potential longer-term resource use, productivity effects, or medication and other healthcare resource use changes. These omissions were deliberate, consistent with the early-phase, proof-of-concept nature of the evaluation, but they highlight the need for future analyses to extend the model to longer time horizons, to incorporate QALY outcomes, and to include real-world follow-up data on the durability of metabolic improvements.

## CONCLUSION

In summary, the present modeling analysis provides quantitative evidence that short-term, intensive weight-loss and lifestyle intervention can translate into meaningful relative reductions in predicted diabetes-related complication risk, even within a 3-month time frame. By combining empirically observed changes in HbA1c, body weight, and blood pressure with a validated outcomes model, the study demonstrates that early metabolic improvements can be expressed not only in clinical but also in economic terms through a pragmatic short-horizon efficiency metric.

The findings reinforce that the magnitude of initial change matters: reductions of approximately 1 percentage point in HbA1c and around 10 kg in body weight correspond to clinically meaningful thresholds known to influence long-term outcomes. If maintained, such improvements would be expected to yield substantial absolute event reductions and downstream cost savings. The analysis therefore highlights the potential of structured, scalable programs like RESET to deliver rapid improvements in modeled risk factors and projected complication risk at modest cost, while longer-term controlled evidence is needed before drawing conclusions about sustained clinical benefit or routine implementation.

At the same time, these results should be interpreted as proof-of-concept rather than definitive cost-effectiveness estimates. Long-term follow-up and extended modeling, incorporating sustained metabolic trajectories, QALYs, and downstream cost offsets, are warranted to establish the durability and full economic impact of these early benefits. Future work will extend the present framework to longer horizons and assess how maintenance of risk-factor improvements influences lifetime outcomes and cost-utility.

Taken together, the study illustrates how short-term clinical data can be leveraged through validated modeling to provide an early modeled signal of potential patient-relevant and economic value. Maintaining the observed improvements in glycaemia, weight, and blood pressure beyond the weight-loss phase remains the critical next step toward realizing their long-term potential for both individuals with type 2 diabetes and the wider health system.

### Disclosures

B.S. received consulting fees from Abbott Nutrition for conducting this analysis. K.W.K., R.R., M.C.-R., and S.S. are employees of Abbott Nutrition. N.N., C.L., and L.T. are employees of Changing Health Ltd.

## Supplementary Material

Online Supplementary Material

## References

[1] Ong K. L., Stafford L. K., McLaughlin S. A.. (2023). Global, regional, and national burden of diabetes from 1990 to 2021, with projections of prevalence to 2050: a systematic analysis for the Global Burden of Disease Study 2021. Lancet.

[2] Huang X., Wu Y., Ni Y., Xu H., He Y. (2025). Global, regional, and national burden of type 2 diabetes mellitus caused by high BMI from 1990 to 2021, and forecasts to 2045: analysis from the global burden of disease study 2021. Front Public Health.

[3] Rodriguez P., San Martin V. T., Pantalone K. M. (2024). Therapeutic inertia in the management of type 2 diabetes: a narrative review. Diabetes Ther.

[4] Louie J. Z., Shiffman D., Rowland C. M.. (2024). Predictors of lack of glycemic control in persons with type 2 diabetes. Clin Diabetes Endocrinol.

[5] Gregg E. W., Chen H., Wagenknecht L. E.. (2012). Association of an Intensive lifestyle intervention with remission of type 2 diabetes. JAMA.

[6] Lean M. E., Leslie W. S., Barnes A. C.. (2018). Primary care-led weight management for remission of type 2 diabetes (DiRECT): an open-label, cluster-randomised trial. Lancet.

[7] Lim E. L., Hollingsworth K. G., Aribisala B. S., Chen M. J., Mathers J. C., Taylor R. (2011). Reversal of type 2 diabetes: normalisation of beta cell function in association with decreased pancreas and liver triacylglycerol. Diabetologia.

[8] UK Prospective Diabetes Study (UKPDS) Group (1998). Intensive blood-glucose control with sulphonylureas or insulin compared with conventional treatment and risk of complications in patients with type 2 diabetes (UKPDS 33). Lancet.

[9] UKPDS investigators (1991). UK Prospective Diabetes Study (UKPDS). VIII. Study design, progress and performance. Diabetologia.

[10] Hayes A. J., Leal J., Gray A. M., Holman R. R., Clarke P. M. (2013). UKPDS outcomes model 2: a new version of a model to simulate lifetime health outcomes of patients with type 2 diabetes mellitus using data from the 30 year United Kingdom Prospective Diabetes Study: UKPDS 82. Diabetologia.

[11] National Institute for Health and Care Excellence (NICE) Type 2 diabetes in adults - management (update): methods, evidence and recommendations for health economic modelling.

[12] Antoniou M., Mateus C., Hollingsworth B., Titman A. (2024). A systematic review of methodologies used in models of the treatment of diabetes mellitus. Pharmacoeconomics.

[13] Yi Y., Philips Z., Bergman G., Burslem K. (2010). Economic models in type 2 diabetes. Curr Med Res Opin.

[14] Trenell M., Taylor L. C., Kerr K. W., Rueda R., Sulo S. (2024). 1836-LB: Real-world impact of a remote, digitally enabled lifestyle program with diabetes-specific formula as part of low-calorie diet among people with obesity and type 2 diabetes—the RESET Study. Diabetes.

[15] Trenell M., Camprubi-Robles M., Taylor L.. (2025). A pathway to type 2 diabetes remission weight reduction and blood glucose improvements are facilitated by a low-calorie diet including diabetes-specific nutritional formula and the use of digitally-enabled reinforcement. Clinical Diabetes.

[16] Clarke P. M., Gray A. M., Briggs A.. (2004). A model to estimate the lifetime health outcomes of patients with type 2 diabetes: the United Kingdom Prospective Diabetes Study (UKPDS) Outcomes Model (UKPDS no. 68). Diabetologia.

[17] McEwan P., Ward T., Bennett H., Bergenheim K. (2015). Validation of the UKPDS 82 risk equations within the Cardiff Diabetes Model. Cost Eff Resour Alloc.

[18] Davis T. M., Cull C. A., Holman R. R. (2001). Relationship between ethnicity and glycemic control, lipid profiles, and blood pressure during the first 9 years of type 2 diabetes: UK Prospective Diabetes Study (UKPDS 55). Diabetes Care.

[19] Leal J., Alva M., Gregory V.. (2021). Estimating risk factor progression equations for the UKPDS Outcomes Model 2 (UKPDS 90). Diabet Med.

[20] (1998). Tight blood pressure control and risk of macrovascular and microvascular complications in type 2 diabetes: UKPDS 38. UK Prospective Diabetes Study Group. BMJ.

[21] Lean M. E. J., Leslie W. S., Barnes A. C.. (2019). Durability of a primary care-led weight-management intervention for remission of type 2 diabetes: 2-year results of the DiRECT open-label, cluster-randomised trial. Lancet Diabetes Endocrinol.

[22] Lean M. E., Leslie W. S., Barnes A. C.. (2024). 5-year follow-up of the randomised Diabetes Remission Clinical Trial (DiRECT) of continued support for weight loss maintenance in the UK: an extension study. Lancet Diabetes Endocrinol.

[23] Wing R. R. (2010). Long-term effects of a lifestyle intervention on weight and cardiovascular risk factors in individuals with type 2 diabetes mellitus: four-year results of the Look AHEAD trial. Arch Intern Med.

[24] Stratton I. M., Adler A. I., Neil H. A.. (2000). Association of glycaemia with macrovascular and microvascular complications of type 2 diabetes (UKPDS 35): prospective observational study. BMJ.

[25] Selvin E., Steffes M. W., Zhu H.. (2010). Glycated hemoglobin, diabetes, and cardiovascular risk in nondiabetic adults. N Engl J Med.

[26] Holman R. R., Paul S. K., Bethel M. A., Matthews D. R., Neil H. A. (2008). 10-year follow-up of intensive glucose control in type 2 diabetes. N Engl J Med.

[27] Wing R. R., Lang W., Wadden T. A.. (2011). Benefits of modest weight loss in improving cardiovascular risk factors in overweight and obese individuals with type 2 diabetes. Diabetes Care.

[28] Zhong Y., Lin P.-J., Cohen J. T., Winn A. N., Neumann P. J. (2015). Cost-Utility Analyses in Diabetes: A Systematic Review and Implications from Real-World Evidence. Value Health.

[29] Drummond M. (2005). Methods for the Economic Evaluation of Health Care Programmes.

[30] O'Mahony J. F., Newall A. T., van Rosmalen J. (2015). Dealing with time in health economic evaluation: methodological issues and recommendations for practice. Pharmacoeconomics.

[31] Willis M., Fridhammar A., Gundgaard J., Nilsson A., Johansen P. (2020). Comparing the cohort and micro-simulation modeling approaches in cost-effectiveness modeling of type 2 diabetes mellitus: a case study of the IHE Diabetes Cohort Model and the Economics and Health Outcomes Model of T2DM. PharmacoEconomics.

[32] Look AHEAD investigators (2014). Eight-year weight losses with an intensive lifestyle intervention: the look AHEAD study. Obesity (Silver Spring).

[33] National Institute for Health and Care Excellence (NICE) Type 2 diabetes in adults: management (NICE guideline NG28).

