# Supplementary material for: Projected Reduction of Diabetes- and Obesity-Related Complication Risks Following the 12-Week Weight-Loss Phase of the RESET Study
Source: J Health Econ Outcomes Res. 2026 Jun 16;13(1):255–63. doi: 10.36469/001c.162856 (PMC13278613; doi:10.36469/001c.162856)
Supplement: Online Supplementary Material [file jheor_2026_13_1_162856_349893.pdf]

Journal of Health Economics and Outcomes Research

Online Supplementary Material

Projected Reduction of Diabetes- and Obesity-Related Complication Risks Following the 12-week Weight-Loss Phase of the RESET Trial. *JHEOR*. 2026;13(1):255-263. doi:10.36469/jheor.2026.162856

Table S1: Projected Event Counts per 1000 Patients at Baseline and After the 12-Week Intervention

This supplementary material has been provided by the authors to give readers additional information about their work.

This is an open-access article distributed under the terms of the Creative Commons Attribution 4.0 International License (CCBY-4.0). View this license's legal deed at http://creativecommons.org/licenses/by/4.0 and legal code at http://creativecommons.org/licenses/by/4.0/legalcode for more information.

Table 1. Projected Event Counts per 1000 Patients at Baseline and After the 12-Week Intervention

Event Category	Baseline Events per 1000 Patients	Post-intervention Events per 1000 Patients	Absolute Reduction (Events/1000)	Relative Risk Reduction (%)
Congestive heart failure	2.34	1.79	0.55	–23.4
Ischemic heart disease	2.17	2.11	0.06	–2.6
Myocardial infarction	1.61	1.43	0.18	–10.9
Stroke	2.12	1.79	0.33	–15.5
All macrovascular events	8.23	7.13	1.10	–13.5
Blindness	0.85	0.69	0.16	–18.3
Foot ulcer	0.81	0.57	0.24	–29.9
Amputation	0.46	0.34	0.12	–24.9
Renal disease	0.20	0.16	0.04	–21.2
All microvascular events	2.32	1.77	0.55	–23.9
Total events (all categories)	11.98	10.13	1.85	–15.4
